# Racial and Ethnic Disparities in Opioid Prescriptions for Patients with Abdominal Pain: Analysis of the National Ambulatory Medical Care Survey

**DOI:** 10.3390/jcm12155030

**Published:** 2023-07-31

**Authors:** Awais Ahmed, Nicole McHenry, Shivani Gulati, Ishani Shah, Sunil G. Sheth

**Affiliations:** 1 Department of Medicine, Division of Gastroenterology, Beth Israel Deaconess Medical Center, Harvard Medical School, 330 Brookline Avenue, Rabb 423, Boston, MA 02215, USA; awais32@hotmail.com (A.A.); nicolemchenry9@gmail.com (N.M.);; 2 Department of Internal Medicine, Division of Gastroenterology, University of Utah Hospital, 50 N Medical Drive, Salt Lake City, UT 84132, USA

**Keywords:** ethnic disparities, opioid prescriptions, inequities, abdominal pain, pain management

## Abstract

Background: Disparities in pain control have been extensively studied in the hospital setting, but less is known regarding the racial/ethnic disparities in opioid prescriptions for patients with abdominal pain in ambulatory clinics. Methods: We examined opioid prescriptions during visits by patients presenting with abdominal pain between the years of 2006 and 2015, respectively, in the National Ambulatory Medical Care Survey database. Data weights for national-level estimates were applied. Results: We identified 4006 outpatient visits, equivalent to 114 million weighted visits. Rates of opioid use was highest among non-Hispanic White patients (12%), and then non-Hispanic Black patients (11%), and was the lowest in Hispanic patients (6%). Hispanic patients had lower odds of receiving opioid prescriptions compared to non-Hispanic White patients (OR = 0.49; 95% CI, 0.31–0.77, *p* = 0.002) and all non-Hispanic patients (OR 0.48; 95% CI 0.30–0.75; *p* = 0.002). No significant differences were noted in non-opioid analgesia prescriptions (*p* = 0.507). A higher frequency of anti-depressants/anti-psychotic prescriptions and alcohol use was recorded amongst the non-Hispanic patients (*p* = 0.027 and *p* = 0.001, respectively). Conclusions: Rates of opioid prescriptions for abdominal pain patients were substantially lower for the Hispanic patients compared with the non-Hispanic patients, despite having a decreased rate of high-risk features, such as alcohol use and depression. The root cause of this disparity needs further research to ensure equitable access to pain management.

## 1. Introduction

The US Institute of Medicine’s report in 2003 titled “*Unequal Treatment: Confronting Racial and Ethnic Disparities in Health Care*” examined the extent and causes of racial and ethnic disparities in healthcare across the United States (US) [1]. It concluded that racial and ethnic minorities in the US receive lower quality healthcare than non-minorities, even when controlling for factors, such as insurance status, income, education, access, and patient preferences.

One area in which these disparities have been particularly pronounced is the prescribing practices of opioids for pain control [2]. Opioids are commonly prescribed to manage moderate-to-severe pain for a variety of causes, both benign and malignant. Disparities in pain control, studied extensively in the acute setting, have demonstrated that opioid prescriptions are less likely to be prescribed to racial–ethnic minorities for a range of conditions. Studies which have assessed patients in the acute setting, like the emergency department (ED), have consistently shown that non-Hispanic Whites are more likely to receive opioids for pain management compared to the non-Hispanic Black and Hispanic populations in spite of being under similar clinical situations, perceptions of pain, and expectations for pain control [3,4,5,6]. These disparities in opioid prescribing practices contribute to inadequate pain management, decreased quality of life, and increased healthcare costs due to psychological effects, risk of developing chronic pain, and economic instability [7,8,9].

Less is known about the racial and ethnic disparities in opioid prescription patterns in the ambulatory setting. A small number of older studies have reported that ethnic minority patients, especially Hispanics, are less likely to receive opioid medication by primary care physicians for acute and chronic pain of various etiologies [10,11]. Reasons to explain these discrepancies are thought to be multifactorial, including physician bias, language barrier, inadequate follow up, and issues pertaining to healthcare access. More recent studies evaluating the evolution of opioid prescribing patterns are lacking, and in times of heightened awareness around the opioid epidemic in the US, it would be noteworthy to assess whether racial and ethnic disparities persist [12].

Abdominal pain is one of the most common causes of healthcare visits in the US, for which there has been an increase in opioid prescribing patterns for its treatment [13,14]. Hence, it affords the ability to investigate the opioid prescribing patterns in the outpatient setting across a broad population over a commonly encountered diagnosis. However, there is limited data available that have evaluated the racial and ethnic disparities in this population. We have previously shown that outpatient visits by Hispanic patients were less likely to record opioid prescriptions when compared to visits by non-Hispanic Blacks and Whites for pain control in benign pancreatic disease [15].

Identifying and understanding the factors contributing to outpatient opioid prescription disparities is crucial to ensure equitable access to appropriate pain management for patients, regardless of their race or ethnicity. To that end, we elected to analyze data from the National Ambulatory Medical Care Survey (NAMCS). The NAMCS dataset is ideal for researching outpatient prescription disparities as it is built on a thorough, annual, and nationally representative survey of ambulatory care visits in the US. With a focus on care provided by generalist clinicians, the NAMCS dataset provides a unique insight into abdominal pain management with opioids outside specialty care settings. Our paper aimed to examine the extent of racial–ethnic disparities in opioid prescriptions in the outpatient treatment of abdominal pain using NAMCS data from the period of 2006–2015.

## 2. Methods

### 2.1. Data Source

Data were extracted from the 2006 to 2015 editions of the National Ambulatory Medical Care Survey (NAMCS), an annual National Center for Health Statistics (NCHS) survey. This survey was distributed to achieve a nationally representative sample of ambulatory care appointments with non-federally employed clinicians using a multi-stage probability sampling design based on geography, physician specialty, and individual patient visits. Within participating medical offices, a systematic random sample of patient visits over a 1 week period were selected for data collection. Information provided from these patients via a standardized patient record form included patient demographics; diagnosis codes (of up to five) using the *International Classification of Diseases, Ninth Revision, Clinical Modification* (ICD-9-CM); NCHS Reason for Visit Classification for Ambulatory Care codes (of up to three), and medications (of up to thirty) listed using the Cerner Multum Lexicon Plus drug database.

### 2.2. Patient Visits

We identified visits made by adult patients (age ≥ 18) presenting for abdominal pain with general internists based on the NCHS Reason for Visit Classification code 1545 (“abdominal pain”). We limited our analysis to patients with abdominal pain as the primary reason for their visit.

### 2.3. Outcomes and Covariates

The primary outcome of interest was new or continued opioid analgesic prescription. Narcotic analgesic and narcotic analgesic combination prescriptions were identified using the Multum Therapeutic Classification categories (Level 1: 057, Level 2: 058, and Level 3: 060 and 191, respectively). To identify the specific opioid analgesic medications, we used the NCHS five-digit drug codes.

Covariables included sex (male and female), age, and race/ethnicity (non-Hispanic White, non-Hispanic Black, Hispanic, and non-Hispanic other). In addition, we identified non-opioid analgesia prescriptions, including non-steroidal anti-inflammatory drugs, salicylates, analgesic combinations, and cox-2 inhibitors (Level 1: 057, Level 2: 058, and Level 3: 061, 062, 063, and 278, respectively). Available data for comorbid conditions, such as smoking, alcohol use, depression, addiction (available from 2014 onwards), as well as insurance status, were also collected. Data on anxiety history was not available in this dataset. The major reason for visit (variable MAJOR), as determined by the provider at the visit, was used to compare the nature of the visits themselves (new problem, chronic problem, pre or post-surgical follow up, and preventive care visit).

### 2.4. Analysis

Statistical tests included the *t*-test, adjusted Wald test, chi-square analysis, and bivariate and multivariate logistic regression, as appropriate, to compare the outcomes between these racial–ethnic groups. Visits by female patients or non-Hispanic White patients were used as the reference group. Odds ratios (OR) were expressed with 95% confidence intervals (CI), and *p* values less than 0.05 were considered to be statistically significant.

We applied standardized NCHS-provided data weights to achieve nationally representative estimates. For weighted analysis, we followed the NAMCS guidelines requiring a minimum of 30 unweighted visits per subgroup and a relative standard error of 30% or less. Analysis was conducted using Stata version 14 (StataCorp, College Station, TX, USA). No institutional review board approval was required for this publicly available, and de-identified database analysis.

## 3. Results

### 3.1. Study Population

Between the years of 2006 and 2015, respectively, we identified 4814 ambulatory general/primary care visits with abdominal pain as the primary reason for visit. Excluding 808 visits that were made by pediatric patients, we identified a total of 4006 visits, corresponding to 114.0 million weighted visits. The majority of visits involved non-Hispanic White patients (74.3 million), followed by Hispanic (21.0 million), non-Hispanic Black (11.7 million), and non-Hispanic other patients (7.0 million). More visits involved female patients (74.2 million) compared to male patients (39.7 million).

### 3.2. Opioid Prescriptions

Opioid prescriptions were recorded in 13% of unweighted visits and 11% of weighted visits, respectively (Table 1). The type of opioids and the unweighted number of visits at which they were prescribed are provided in Table 2. The two most commonly prescribed opioids were the narcotic analgesia combination medications hydrocodone–acetaminophen and oxycodone–acetaminophen. Together, these two medications accounted for 48% of all unweighted opioid prescriptions.

Opioid prescription rates were the highest at visits for abdominal pain made by non-Hispanic White patients (12%), followed by visits from non-Hispanic Black patients (11%), and were lowest among visits by Hispanic patients (6%). Multivariate logistic regression demonstrated that only visits made by Hispanic patients were less likely to record opioid prescriptions than those of non-Hispanic White patients (OR = 0.49; 95% CI, 0.31–0.77, *p* = 0.002) (Table 1). Bivariate logistic regression also demonstrated a lower opioid prescription rate at visits by Hispanic patients compared to all non-Hispanic abdominal pain patients (OR 0.48; 95% CI 0.30–0.75; *p* = 0.002). There was no significant difference observed in the opioid prescription rates for abdominal pain visits by sex (10% vs. 11%, respectively, *p* = 0.54).

### 3.3. Demographics, Covariates, and Abdominal Pain

The average patient age was similar among visits by all racial–ethnic groups (non-Hispanic White 51.5, Non-Hispanic Black 49.9, Hispanic 51.2, and non-Hispanic other 50.2, respectively; OR 1.12 95% CI 0.79–1.57; *p* = 0.833). Table 3.

There was no significant difference noted in non-opioid analgesia prescriptions (non-Hispanic White 16.4%, non-Hispanic Black 19.0%, Hispanic 21.5%, and non-Hispanic other 21.0%, respectively; *p* = 0.507). The rates of depression diagnosis were found to be similar at visits by non-Hispanic and Hispanic patients (10.9% vs. 7.5%, respectively; *p* = 0.072); however, there was a higher number of anti-depressants/anti-psychotic prescriptions reported amongst the non-Hispanic patients (*p* = 0.027).

Data on alcohol and substance abuse was available from the year of 2014 onwards. Among the 22.3 million weighted visits in 2014 and 2015, there was a higher reported level of alcohol abuse amongst visits by non-Hispanic patients compared to Hispanic patients (2.9% vs. 0.2%, respectively, *p* = 0.01). Conversely, there was a similar prevalence of smoking (non-Hispanic White 19.3%, non-Hispanic Black 21.7%, Hispanic 12.7%, and non-Hispanic other 11.0%, respectively, *p* = 0.073) and substance abuse history (non-Hispanic White 1.1%, non-Hispanic Black 0.2%, Hispanic 0.4%, and non-Hispanic other 0%, respectively, *p* = 0.432) across visits that were made by all racial–ethnic groups.

Stratifying insurance types revealed that visits made by Hispanic patients were significantly more likely to be self-pay visits (or no recorded insurance) compared to visits made by non-Hispanic patients (8.3% vs. 2.9%, respectively, *p* = 0.008). This difference persisted between visits by Hispanic patients, non-Hispanic Black patients, and other minority patients (8.3% vs. 2.4% and 2.3%, respectively, *p* = 0.003). A similar trend was noted in workers’ compensation visits for Hispanic vs. non-Hispanic patients (0.5% vs. <0.01%, respectively). Insurance visits covered by private insurance, Medicare, and Medicaid were found to be similar across all groups.

The most common diagnoses of abdominal pain among those prescribed opioids were unspecified abdominal pain, right upper quadrant pain, esophageal reflux, calculus of gallbladder, chronic pain, right lower quadrant pain, and constipation. The major reason for visit was similar among visits by all racial–ethnic groups, with acute concerns accounting for approximately two-thirds of visits by each racial–ethnic group (*p* = 0.781).

## 4. Discussion

We analyzed 114 million weighted outpatient visits for opioid prescription rates among abdominal pain patients by racial–ethnic group. Our dataset was weighted to achieve a nationally representative population, with racial–ethnic group proportions closely matching those of the US Census data [16]. We report significant disparities, with Hispanic patients less likely to receive opioid prescriptions compared to non-Hispanic White patients, followed by non-Hispanic Black patients. This disparity persisted in spite of adjusting for age, sex, non-opioid analgesics use, smoking, alcohol use, chronicity of abdominal pain, and insurance coverage. In the visits analyzed in our study, nearly two-thirds of the patients were female. This result is consistent with the higher proportion of visits by female patients reported in the NAMCS overall. Moreover, this result is unsurprising given that women are at a higher risk for painful conditions [17].

Ly et al. revealed that Hispanic patients were 6.3% less likely than non-Hispanic White patients to receive opioids for abdominal pain and were even less likely to receive opioids for back pain [18]. They also noted that visits for back pain were shorter for Hispanic patients than White patients, leaving less time for evaluation. In contrast to this study, we used the NAMCS database and applied standard weights and measures to extrapolate a more substantive representation of the US population, with the primary outcome focused on opioid prescription for abdominal pain, as well as evaluating confounding factors that may impact prescribing patterns, such as substance abuse, alcohol use, smoking, depression, and the use of non-opioid analgesia and neuromodulators.

Another study conducted at a safety net hospital demonstrated that White patients were in the 95th percentile of receiving opioid prescriptions compared to racial–ethnic minorities [19]. The study also noted that White patients were more likely to experience an opioid use disorder subsequent to an opioid prescription compared to minority patients. This finding challenges clinician misconceptions and bias, which leans towards higher concerns of opioid abuse among minority patients, leading to persistent discrepancies in pain management strategies between White and minority patients [20,21]. This possible bias exists in spite of the fact that between the years of 1990 and 2010, respectively, the rate of opioid-related mortality for Whites increased by 225% and decreased for Blacks and Latinos by 3% and 17%, respectively [22]. These inherent clinician biases are in part driven by certain risk factors attributed to higher odds of an opioid use disorder, including mental health illness, history of substance abuse, and alcohol and tobacco use disorders [23,24]. However, in our study, despite a higher prevalence of anti-depressants/anxiolytics and alcohol use in non-Hispanics, opioid prescriptions favored non-Hispanic Whites, which suggests an inherent bias in prescribing opioids for minorities, specifically for Hispanics.

As we have previously explored in regard to opioid prescription disparities in pancreatic disease [15], provider bias may not fully account for racial–ethnic differences in opioid prescriptions. Broader social, environmental, and economic factors may also explain the disparities seen in our analysis.

Lack of healthcare access and insurance has been one such barrier cited in understanding the pain control disparities amongst minorities [25]. In our study, Hispanics had the highest rate of self-pay visits, which is reflective of Hispanics being the largest uninsured ethnic group in the US according to the US census of 2021 [26,27]. Lack of insurance coverage may contribute to hesitation by providers and Hispanic patients to pursue treatments, like opioids, that require frequent visits and monitoring. Accessibility issues may be compounded by a lack of opioid supplies in Hispanic neighborhoods. Although recent data is lacking, two older studies demonstrated that pharmacies were less likely to have adequate opioid supplies if they were located in minority communities than in White communities. Without easy physical access, Hispanic patients and their providers could opt out of opioid treatment as a consequence. However, rates of non-opioid analgesia prescriptions were not found to be significantly higher at visits made by Hispanic patients in our study, suggesting that access to opioids and related care were not the primary causes of this disparity. Language and cultural preferences may also contribute to opioid prescription disparities in the Hispanic community. A systematic review of studies evaluating ethnic differences in the pain response demonstrated that pain evaluation, coping strategies, and treatment preferences differed among ethnic groups [28]. Spanish-speaking Hispanics face communication challenges and patient–provider relationship building challenges not faced by English-speaking patients. Additionally, Spanish-speaking Hispanics may have even lower insurance rates than non-Spanish speakers and prefer self-management and nonpharmacological treatments over medication [26,29]. These trends were each reflected in our analysis regarding insurance and medication use. Within Hispanic communities, cultural beliefs about stoicism and stigma regarding addiction and mental illness have also been shown to contribute to a reduced opioid use [30,31,32].

Our study has a number of strengths, most notably that of the representative population studied. Through the NAMCS database, intentional oversampling and weight enhanced visits (amounting to 114 million visits) extrapolated a large representative population to study outcomes. The ability to isolate exact diagnoses based on ICD coding, demographics, and co-variates allows for curating a clean cohort of patients to study, which included adjusting for pain chronicity, mental health, alcohol use, tobacco use, substance abuse, and insurance carriers.

However, there are certain limitations as well. Firstly, the subjective discussions of the provider–patient relationship, which go into shared decision making, has not been accounted for in this analysis. For example, cultural differences in the perception and management of pain varies significantly between ethnicities. Hispanics and other minorities may favor non-opioid analgesia and self-care over opioid use [30,32]. Furthermore, characteristics of abdominal pain beyond acuity was not discernable in our analysis. The etiology of abdominal pain has a broad differential, from the benign functional bowel disorder to malignant disease, and the decision to treat pain with opioids is a multifaceted process, which includes history, physical examination, and diagnostic evaluation. Furthermore, the current guidelines do not support nor provide guidelines for the opioid-based treatment of non-malignant pain and may be variably influenced by disease characteristics. For example, in another NAMCS-based study, racial and ethnic disparities were seen in benign pancreatic disease, but not in malignant pancreatic disease, which points to a more standardized approach to pain management in malignant disease which potentially reduces biases [15].

In conclusion, racial and ethnic disparities in pain management persists in the treatment of abdominal pain, with lower rates of opioid-based analgesia among Hispanics. The reason for this disparity remains challenging to identify, and is a complex issue with numerous causes, including provider biases. Understanding and addressing these disparities is crucial in improving healthcare equity and in providing appropriate pain management for all patients.

## Figures and Tables

**Table 1:** Weighted opioid prescriptions by sex and racial–ethnic group at visits for adult patients with a primary reason for visit of abdominal pain reported in the NAMCS during the period of 2006–2015. Numbers are reported in millions.

		No Opioid	Opioid Prescription	OR (95% CI)	*p*-Value
		N = 101.71 (89%)	N = 12.24 (11%)		
Race/Ethnicity, N (%)					
(A)	Non-Hispanic	82.0 (88)	11.0 (12)	Reference	Reference
	Hispanic	19.7 (94)	1.26 (6)	**0.48 (0.30–0.75)**	**0.002**
(B)	White non-Hispanic	65.7 (88)	8.58 (12)	Reference	Reference
	Black non-Hispanic	10.4 (89)	1.3 (11)	0.98 (0.57–1.69)	0.94
	Hispanic	19.7 (94)	1.26 (6)	**0.49 (0.31–0.77)**	**0.002**
	Other non-Hispanic	5.9 (84)	1.1 (16)	1.41 (0.51–3.93) *	0.51 *
Sex, %					
	Female	66.5 (90)	7.7 (10)	Reference	Reference
	Male	35.2 (89)	4.5 (11)	1.12 (0.79–1.57)	0.54

* Relative Standard Error = 52%.

**Table 2:** Opioid medications prescribed at visits by adult patients with a primary reason for visit of abdominal pain reported in the NAMCS during the period of 2006–2015.

Opioid Medication	Frequency (n)
Hydrocodone–acetaminophen	210
Oxycodone–acetaminophen	68
Oxycodone	64
Tramadol	63
Hydrocodone	36
Codeine–acetaminophen	26
Fentanyl	23
Propoxyphene–acetaminophen	20
Morphine	19
Hydromorphone	17
Meperidine	13
Methadone	10
Oxymorphone	3
Hydrocodone bitartrate–acetaminophen	2
Nalbuphine hydrochloride	2
Butorphanol	1
Codeine–butalbital	1
Codeine–guaifenesin	1
Dihydrocodeine–acetaminophen–caffeine	1
Hydrocodone–homatropine	1
Hydromorphone–promethazine	1
Paregoric	1
Propoxyphene napsylate	1
Tapentadol	1

**Table 3:** Demographic factors, comorbidities, medication usage, and visit data at visits by adult patients with a primary reason for visit of abdominal pain in NAMCS 2006–2015.

	Non-Hispanic	Hispanic	*p*-Value	Non-Hispanic White	Non-Hispanic Black	Hispanic	Non-Hispanic Other	*p*-Value
**Age**	51.2	51.2	0.974	51.5	49.9	51.2	50.2	0.833
Non-opioid analgesia (%) *	17.1	21.5	0.204	16.4	19.0	21.5	21.0	0.507
**Antidepressants/antipsychotics (%)**	11.6	7.0	**0.027**	11.4	9.9	7.0	16.9	0.178
**Smoking (current) (%) †**	19.0	12.7	0.071	19.3	21.7	12.7	11.0	0.073
**Alcohol abuse (%) §**	2.9	0.2	**0.01**	4.0	0	0.2	0	0.442
**Depression (%)**	10.9	7.5	0.072	11.4	10.3	7.5	6.8	0.082
**Substance abuse (%) ¶**	0.8	0.4	0.338	1.1	0.2	0.4	0	0.432
Insurance (%) **			**0.008**					**0.003**
Private	56.8	54.8		56.9	50.9	54.8	66.5	
Medicare	27.3	22.0		28.6	24.9	22.0	16.4	
Medicaid/CHIP	10.5	11.9		9.2	18.9	11.9	10.4	
Workers’ compensation	<0.01	0.5		<0.01	0.1	0.5	0	
Self-pay	2.9	8.3		3.0	2.4	8.3	2.3	
No charge/charity	0.8	0.9		0.6	1.8	0.9	2.1	
Other	1.7	1.6		1.7	1.0	1.6	2.3	
**Major reason for visit type (%) ††**			0.781					0.914
Acute	62.2	66.0		61.7	66.5	66.0	60.6	
Chronic, routine follow up	15.4	14.2		15.7	12.3	14.2	17.4	
Chronic, flare up	14.7	13.2		14.7	14.8	13.2	15.3	
Pre/post-surgery	5.6	4.3		5.8	5.2	4.3	3.8	
Preventative care	2.1	2.3		2.1	1.2	2.3	3.0	

* Includes nonsteroidal anti-inflammatory drugs, salicylates, analgesic combinations, and cox-2 inhibitors. † Based on 80.1 million weighted visits (2916 unweighted observations). § Based on 22.3 million weighted visits (699 unweighted observations) only from 2014 to 2015, respectively. ¶ Based on 22.3 million weighted visits (699 unweighted observations) only from 2014 to 2015, respectively. Does not meet the NAMCS criteria for 30 patients per subgroup as required for reliable weighted analysis (only 11 patients were recorded to have substance abuse). ** Based on 109.8 million weighted visits (3793 unweighted observations). †† Based on 111.5 million weighted visits (3939 unweighted observations).

## Data Availability

Publicly available datasets were analyzed in this study. This data can be found here: ftp.cdc.gov/pub/Health_Statistics/NCHS/Datasets/NAMCS/ (accessed on 4 October 2022).
